# Supplementary material for: Where Is Ethology Heading? An Invitation for Collective Metadisciplinary Discussion
Source: Animals (Basel). 2021 Aug 27;11(9):2520. doi: 10.3390/ani11092520 (PMC8472011; doi:10.3390/ani11092520)
Supplement: Supplementary file 1 [file animals-11-02520-s001.zip › supplementary- animals-1225637.pdf]

ANNEX. Online questionnaire
Link: shorturl.at/uKLRT

I. Demographic data

Q1: In which continent were you born?

R1: Africa/Asia/Central or South America/North America or Canada/ Europe/ Oceania

Q2: In which continent is your current institution placed?

R2: Africa/Asia/Central or South America/North America or Canada/ Europe/ Oceania

Q3: What degree did you study?

R3: Psychology/ Biology or Marine Biology/ Philosophy/ Environmental Studies/ Other

Q4: Optional: How old are you?

Q5: How many years of experience do you have in this area of research?

R5: 0 to 5 years/ 5 to 10 years / 10 to 20 years / more than 20 years

Q6: In which of these sites of research do you commonly conduct your studies? (multiple answer)

1. R6: Lab or Zoo/ Free range or Reserve/ Wild
2. Q7: OPTIONAL: Would you share some email address to contact you (just for sharing the potential published article, not for other purposes)?

II. Our discipline

Q8: How would you name our discipline? Please, choose only one even when many are related to each other or even if there is none that entirely conveys your preference

R8: Animal cognition / Animal behaviour/ Ethology/Comparative Psychology

Q9: Do you think our discipline have some common core definitions that would find little discrepancy among researchers?

R9: Yes, there is consensus in almost all the concepts of our discipline / Yes, but there is consensus in only few concepts / No, there is almost no consensus in the concepts of our discipline

Q10: Please, name some of the concepts you were thinking there is NO consensus on (if you responded there is consensus in almost all concepts, please write "does not apply") (open question)

Q11: Please, name some of the concepts you were thinking there IS consensus on (if you responded there is almost no consensus, please write "does not apply") (open question)

Q12: Would you say there is consensus for each one of these concepts in our field? (a table is displayed to pick "lot of consensus", "Moderate consensus" or "Little consensus")

R12: Cognition / Social Learning / Associative Learning / Tinbergen's questions / Evolution Theory / Culture/ Tool use / Emotion / Basic Psychological Cognitive Capacities / Linguistics / Cooperation / Prosociality

Q13: Which would it be your main underlying interest when doing research in our discipline? I know all may be tempting: try to choose only one!

R13: To know more about X non-human species / To know more about the differences/similarities between X and Y non-human species / To know more about the differences/similarities between X non-human species and humans

III. Species

Q14: Which group of animal you usually study? (I know you may have worked with a bunch of them, please, choose the one you would feel more confident to talk about)

R14: Non-human primates / Dogs and wolves / Birds / Non avian reptiles / Fish or Marine animals / Farm animals (Horses, chicken…) / Invertebrates / Rodents / Wild carnivores (Bear, Hyena…) / Not listed above (you'll be able to specify them in the next question)

Q15: Could you list the name/scientific name of the species you usually study? (open question)

Q16: Why did you choose to study that/those species?

R16: I could investigate more in my research question or it is a model species / Availability, low cost maintenance or sample size / Chance, job opportunity or funding / Fascination about that or those species / It is understudied / It is very skilled

Q17: Have you considered to study another species?

R17: Yes/No

Q18: If you said yes above: which other group of animal would you study? (Please, choose only one)

R18: I responded “no” above / Non-human primates / Dogs and wolves / Birds / Non avian reptiles / Fish or Marine animals / Farm animals (Horses, chicken…) / Invertebrates / Rodents / Wild carnivores (Bear, Hyena…)/ Not listed above (you'll be able to specify them in the next question)
Q19: Could you list the name/scientific name of the species you would also like to study? (if you said "no" above, please write "does not apply") (open question)
Q20: Why would you choose to study that/those species?
R20: I responded “no” above / : I could investigate more in my research question or it is a model species / Availability, low cost maintenance or sample size / Chance, job opportunity or funding / Fascination about that or those species / It is understudied / It is very skilled

IV. Procedures
Q21: Do your studies usually involve ecological apparatuses (elements made only of natural (non synthetic) material that reliably resemble the type of problems the species would find in its natural habitat)?
R21: Yes / No/ Does not apply (because I work in the wild / because I do observational studies, etc)
Q22: Do you know what "umwelt" refers to and do you actively apply it to the design of your studies? (please, if you don't, do not google it after you finish the questionnaire! I'm just wondering)
R22: Yes and I apply it in almost all my studies / Yes but I rarely apply it in my studies / No
Q23: optional: If you answered any of the "yes" previous responses, could you provide and example on how you had applied the umwelt to your studies? (you can write: "it does not apply") (open question)
Q24: How much relevant do you think it is considering these factors when designing an experiment? (a table is displayed to pick “a lot”, “Moderate”, “Low”or “Not relevant at all”)
R24: Species sensory modalities / Species ethogram / Potential sex-behavioural differences / Psychological characteristics (eg. shyness) / Apparatuses resembling nature / Social factors (e.g., rank)
Q25: Have you actively used some technology in your studies? (avoid easy accessible tech such as cameras or stopwatches. I'm thinking of more sophisticated technology, necessary or mandatory to conduct your research)
R25: Yes/ No
Q26: If you said yes, and you work in the lab, please rank the devices according to your use: (a table is displayed to pick “I used it a lot”, “I used it sometimes”, “I do not use it”or “I do not use tech/ I do not work in the lab”)
R26: Touchscreen / Automatized feeders / Artificial chambers/ Eye tracking / ECG or fMRI or Xray / Sound analysis / GPS or Tracking or Telemetry/ OMICs technologies/ Drone / Non-human sensory devices: ultrasounds, etc. / Computer simulations
Q27: If you said yes, and you work in free range/farm/wild, please rank the devices according to your use: (a table is displayed to pick “I used it a lot”, “I used it sometimes”, “I do not use it”or “I do not use tech/ I do not work in these sites”)
R26: Touchscreen / Automatized feeders / Artificial chambers/ Eye tracking / ECG or fMRI or Xray / Sound analysis / GPS or Tracking or Telemetry/ OMICs technologies/ Drone / Non-human sensory devices: ultrasounds, etc. / Computer simulations
Q28: In case the technology devices you usually use was not listed above, could you please specify its name and use? (you can write "does not apply") (open question)
Q29: Dream with me: which technology device would you like to have in your studies and why? (skip this question if it does not apply) (open question)

V. Data analysis
Q30: Which is the common N sample (of non-human animals) you usually have in your studies?
R30: 0 to 7 subjects/ 8 to 12 subjects / 13 to 20 subjects / 21 to 40 subjects / more than 40 subjects
Q31: Which statistic program do you usually use to analyze your data? Please, choose only one
R31: R / SpSS / MATLAB / Python / JAMOVI/ Other
Q32: Which kind of analysis do you usually conduct in your research? You can select more than one (multiple answer)
R32: Descriptive / Non-parametric / Parametric / Correlations / Logistic regression / GLMM / Big data analysis / If it applies, I usually report the statistical power

VI. Networking
Q33: Do you actively engange and have contact with other researchers (that are not your close friends) studying the same species you study and talk about research? I'm thinking of regular conversations not only circumscribed to conferences
R33: Yes, several times each year / Yes, a couple of times each year / No

VII. Past, present, future
Q34: PAST: In your opinion, which events/ideas/inventions do you consider to be groundbreaking/an inflection point in our discipline? (open question)

Q35: PRESENT: In your opinion, which are the main shortcomings/difficulties/obstacles that our discipline currently faces? (open question)
Q36: FUTURE: Which events could have an impact in our discipline in the future? (open question)
Q37: Please, rank how much these events have an impact on our discipline: (a table is displayed to pick “Not much”, “Something”, “much”, “A lot”
R37: Pandemic (COVID-19) / Lack of funding / Low gender or ethnic diversity in research community / Lack of dialogue lab and wild researchers / Lack of interdisciplinarity / Lack of collaboration between researcher groups/ Publish or Perish / Anthropocentrism/ Climate change / Species extinction / Animal welfare and ethics / Reproducibility crisis / Open Science paradigm/ Lack of long-term data (e.g., longitudinal studies)/ Small sample sizes / Robotics or new technology or softwares
Q38: Would you recommend me some readings you think are of interest for this topic? (even if they would seem very remote) (open question)
3. Q39: Any further comment? (open question)
